# Mapping the transcriptomic changes of endothelial compartment in human hippocampus across aging and mild cognitive impairment

**DOI:** 10.1242/bio.057950

**Published:** 2021-05-24

**Authors:** Daniel V. Guebel, Néstor V. Torres, Ángel Acebes

**Affiliations:** 1 Program Agustín de Betancourt, Universidad de La Laguna, Tenerife 38200, Spain; 2 Department of Biochemistry, Cellular Biology and Genetics, Institute of Biomedical Technologies, Universidad de La Laguna, Tenerife 38200, Spain; 3 Department of Basic Medical Sciences, Institute of Biomedical Technologies, University of La Laguna, Tenerife 38200, Spain

**Keywords:** Vascular remodeling, Sprouting angiogenesis, Atherosclerosis, Vascular inflammation, Capillary arterialization, Splice forms, Microarray

## Abstract

Compromise of the vascular system has important consequences on cognitive abilities and neurodegeneration. The identification of the main molecular signatures present in the blood vessels of human hippocampus could provide the basis to understand and tackle these pathologies. As direct vascular experimentation in hippocampus is problematic, we achieved this information by computationally disaggregating publicly available whole microarrays data of human hippocampal homogenates. Three conditions were analyzed: ‘Young Adults’, ‘Aged’, and ‘aged with Mild Cognitive Impairment’ (MCI). The genes identified were contrasted against two independent data-sets. Here we show that the endothelial cells from the Younger Group appeared in an ‘activated stage’. In turn, in the Aged Group, the endothelial cells showed a significant loss of response to shear stress, changes in cell adhesion molecules, increased inflammation, brain-insulin resistance, lipidic alterations, and changes in the extracellular matrix. Some specific changes in the MCI group were also detected. Noticeably, in this study the features arisen from the Aged Group (high tortuosity, increased bifurcations, and smooth muscle proliferation), pose the need for further experimental verification to discern between the occurrence of arteriogenesis and/or vascular remodeling by capillary arterialization.

This article has an associated First Person interview with the first author of the paper.

## Abstract (precis)

**Summary:** An integrative picture about the mechanisms operating in the hippocampal vasculature under normal and pathological scenarios is achieved by the computational dissection of microarray data corresponding to whole tissue samples and focusing on gene splice forms.

## INTRODUCTION

Multiple pieces of evidence point to the incidence of brain vascular disease on cognitive deficits and neurodegeneration (Sweeney et al., 2018; Liu et al., 2018; Baruah et al., 2020). Moreover, this relationship exhibits some common features with systemic pathologies now recognized as risk factors for Alzheimer's disease (Wang et al., 2018; Montagne et al., 2020).

Diagnosis of brain vascular diseases can be reached by several imaging techniques (Computed Tomography Angiography, MRI, PET/SPECT, Transcranial Doppler). Some of these techniques not only provide structural information and detect the injuries in the parenchyma, but allow the quantification of cerebral blood flow (CBF) (Frantellizzi et al., 2020). However, the comprehension of brain vascular pathophysiology requires the understanding of their molecular basis. To achieve this goal, both the multifactorial nature of these diseases and the central concept of neurovascular unity (NVU) should be considered (Takahashi, 2020; Levit et al., 2020). However, the complex biology behind the neurovascular brain pathology is still full of open questions.

Microarray analysis is a relatively simple way to interrogate cells about their transcriptional state (Mantione et al., 2014; Chen et al., 2017). But, to move from the differential detection of some designed probes to the generation of new knowledge about the functional state of the genome in each scenario is a challenging task (Guebel and Torres, 2021). Even the most powerful procedure of RNA-sequencing is not free from limitations (Cao et al., 2018; Boers et al., 2019). Importantly, transcription is not at all equivalent to effective expression, a feature that together with the uncertainty about the post-translational modifications and the unknown identity of the (contextual) interacting partners (including microRNAs and circular RNAs), represents potential limitations to achieve appropriate inferences.

We have previously analyzed a set of published microarray data concerning aging in human hippocampus. By using an optimized algorithm named Q-GDEMAR, we detected many features not evident in their primary analysis. The quantification of the false discovery rate (FDR) is the current way to establish the significance of these findings. Within Q-GDEMAR we have also developed an alternative algorithm to compute the FDR associated to differential genes. A plethora of significant genes and functional dimensions arose from these analyses.

Although the global view achieved has provided new insights to the process of brain aging, we believe that the intricate architecture of these data could still be masking important phenomena. Although obtained from a well-delimited hippocampal region, samples for the microarrays finally end up in a homogenate. Thus, the population of mRNAs under analysis is a mixture that reflects the distinct coexisting cellular populations (neurons, astrocytes, microglia, endothelial cells, mural cells, immune cells, etc.). To overcome this problem, fluorescence-activated cell sorting (FACS) followed by single-cell RNA-sequencing (scRNA-seq) has been implemented. In some cases, transgenic animals were used to produce labelled cells for FACS analysis, but aggressive conditions were required to separate endothelial cells from mural cells (Zhang et al., 2014; Vanlandewijck et al., 2018; Munji et al., 2019). scRNA-seq has also been achieved without transgenic reporters, but the cellular dissociation treatment previous to the *in vitro* labelling for FACS sorting is still necessary (Elahy et al., 2015; Guo et al., 2019; Yousef et al., 2019; Chen et al., 2020; Zhao et al., 2020; Kalucka et al., 2020). The RNA-seq profiling can also be obtained by laser capture microdissection (LCM) (Song et al., 2020). Although LCM provides less distorting conditions, FACS still allows the analysis of most of the cellular populations present in the neurovascular unit. The difficulties to deal with the sub-types of cerebral endothelial compartments is illustrated by the fact that in a new database devoted to endothelial cells (EndoDB, Khan et al., 2019), only 5.4% of its information is in regards to brain endothelial cells.

Given the enumerated problems, we pose that we can exploit the information of ‘global’ microarrays as a proxy, to progress in the comprehension of the hippocampal endothelium compartment. This is accomplished by making a ‘computational deconstruction’ of these microarrays, and establishing which of the analyzed genes could belong to the endothelial cells, based on specific databases. Moreover, to gain depth into their physiological relevance, we will restrict our list to genes belonging to the endothelium class, focusing only on those subsets of genes that being differentials, have splice forms (see Materials and Methods).

In fact, an important fraction of the genome has evolved developing isoforms. Although sometimes the isoforms are redundant, very often distinct isoforms of a given gene deal with different biological functions (Twine et al., 2011; Tollervey et al., 2011; Zhang et al., 2014; Jutzi et al., 2018; Taga et al., 2020). In the last few years, several articles related to splice forms in the brain have been published, focusing mainly on individual molecules (ESR-beta, MHCI, NRXN1, PDE4, AR, MGF, Insulin Receptor, BIN1). However, our approach, rather than based on individual genes/isoforms, attempts to focus on their collective organization in functional networks. In brief, we show here how functional categories related to angiogenesis, which in the global microarray previously appeared as not significant, have been revealed efficiently after our filtering procedure, thus providing new biologically relevant findings.

## RESULTS

### Angiogenesis multidimensionality

**Fig. 1.**
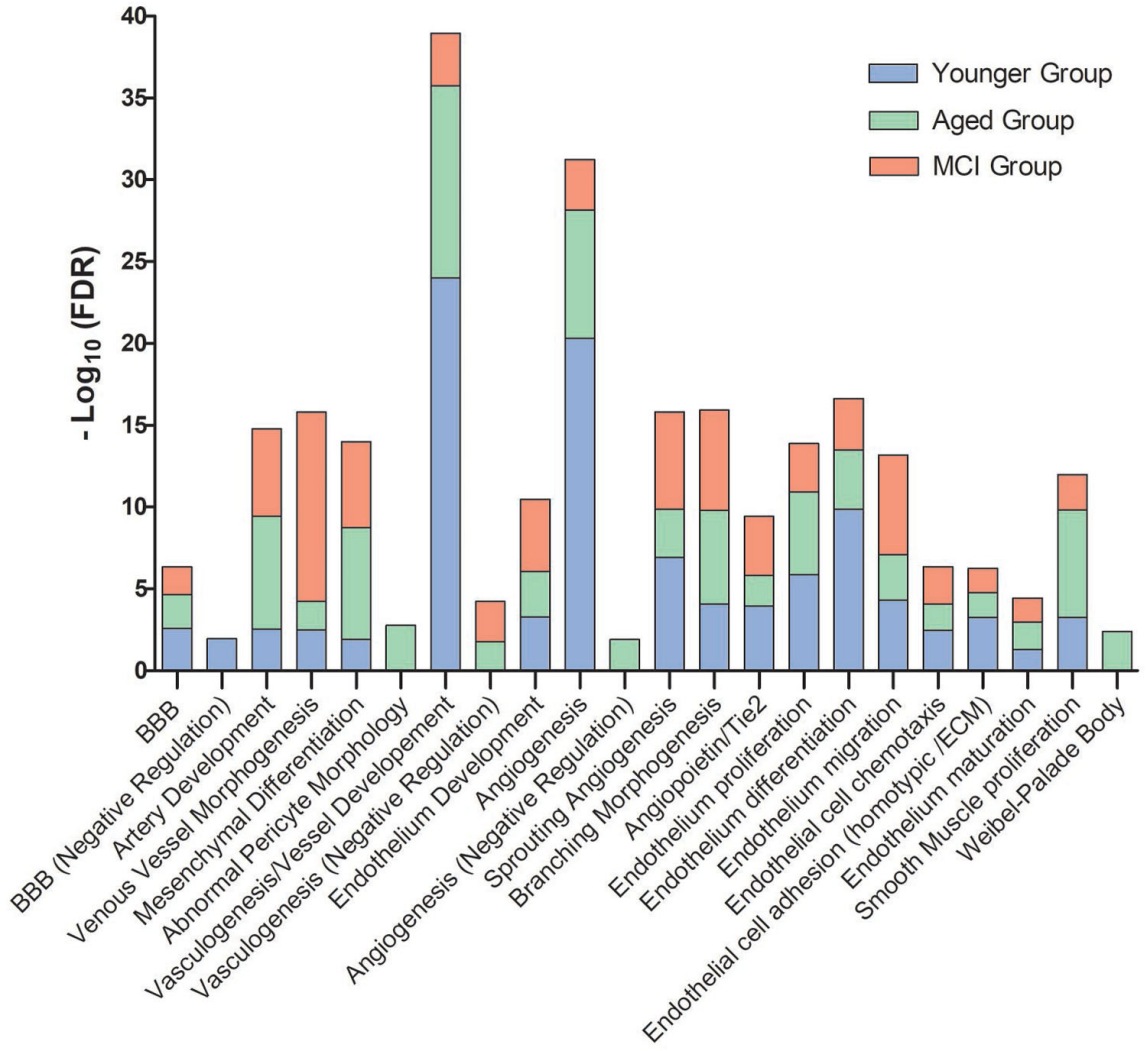
**Comparison of vascular functionalities in human hippocampus across the stages of healthy-adult individuals (Younger Group), healthy-elder individuals (Aged Group), and elder individuals with MCI (MCI Group) by a stacked column representation.** The scale on the y-axis quantifies the ‘strength’ of the significance associated to each feature, computed as -log_10_(FDR), where FDR is the value of false discovery rate. BBB refers to the brain-blood barrier.

Our computational strategy allowed us to detect almost a hundred differential functionalities among the three groups assessed (see Table S1). Some of these functionalities, mainly those related with aspects of angiogenesis, are shown in Fig. 1.

Attempting to explain the physiological basis of the outcomes depicted in Fig. 1, the totality of differential features in Table S1 was grouped according to their patterns of variation. This allowed us to simplify their complex picture down to ten temporal profiles (see Table 1).

**Table 1.**
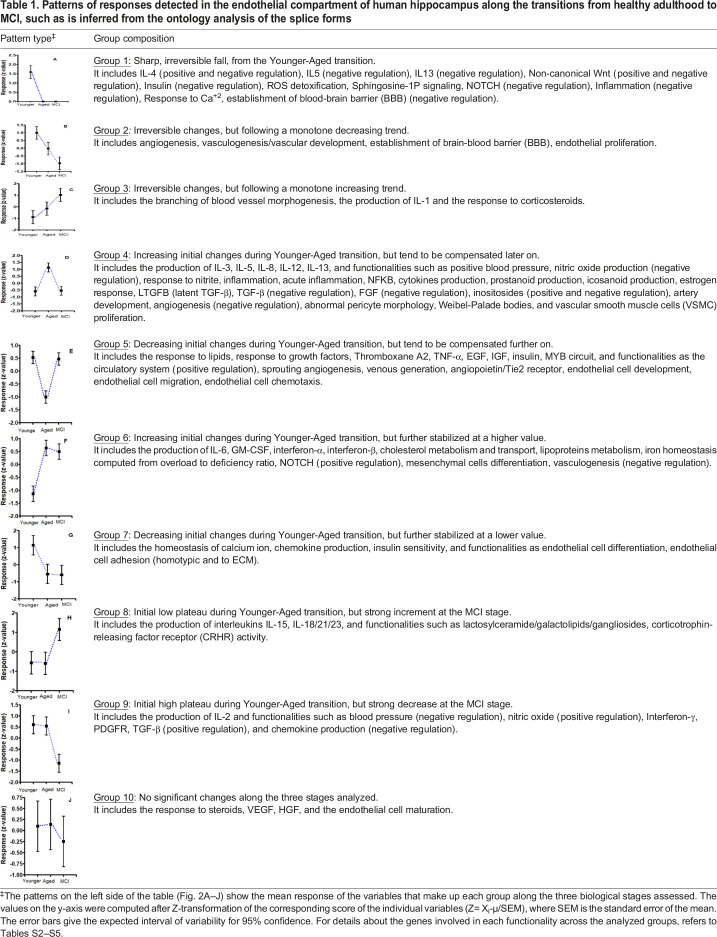
Patterns of responses detected in the endothelial compartment of human hippocampus along the transitions from healthy adulthood to MCI, such as is inferred from the ontology analysis of the splice forms

Table 1 shows that most of the changes in the hippocampal vasculature happened during the transition of the Younger to the Elderly Group. In fact, only two out of ten identified patterns showed changes specifically associated to the MCI condition (see Fig. 2H,I). Moreover, the data showed that angiogenesis implies a series of multidimensional mechanisms.

The ‘establishment of brain-blood barrier’ and ‘angiogenesis’ decreased along the three stages evaluated, running in parallel with ‘vasculogenesis’ and ‘ECs proliferation’ (see Fig. 2B). This differs markedly from both the evolution of ‘ECs migration’ and ‘ECs chemotaxis’ (see Fig. 2E) and the evolution of ‘ECs differentiation’ and ‘ECs adhesion’ (see Fig. 2G). The loss of ‘negative regulation of BBB establishment’ (see Fig. 2A) did not avoid the fall of the ‘positive regulation of the BBB establishment’ (see Fig. 2B). In turn, ‘vascular branching’ showed a completely independent evolution (see Fig. 2C).

In Fig. 2D, ‘artery development’, ‘VSMC proliferation’, ‘IL-8’ and ‘inflammation’ appeared maximized in the Aged Group, and run together as was expected (Kim et al., 2008; Li et al., 2010). They were partially paralleled by the ‘negative regulation of vasculogenesis’ (see Fig. 2F). Conversely, ‘venous generation’ is minimized in the Aged Group, but appeared associated to ‘sprouting angiogenesis’, ‘ECs migration’, and ‘ECs chemotaxis’.

The fact that ‘vasculogenesis’ and ‘endothelial cell development’ in the Younger Group (typical activities in the embryonic stage) appeared as significant as ‘angiogenesis’ (typical of the post-natal stage), suggests that neovascularization occurs through endothelial progenitor cells (EPCs, Malinovskaya et al., 2016). The involvement of EPCs could be possible given their ability for homing and further differentiation (Kioi et al., 2010; Wei et al., 2019). Moreover, endothelial cells could be also generated during neovascularization by trans-differentiation from mesenchymal cells (Balaji et al., 2013) and/or from pericytes (Nwadozi et al., 2020).

### Proliferation/quiescence in hippocampal vasculature

The Younger Group differs from the Aged Group not only in ‘angiogenesis’ (FDR_Younger_=4.9×10^-21^ versus FDR_Aged_=1.5×10^-8^), but also in the ‘endothelins signaling’ (FDR_Younger_=1.9×10^−4^ versus FDR_Aged_=3.8×10^−2^), and in the ‘Glypican 1 circuit’ (FDR_Younger_=2×10^−3^ versus FDR_Aged_=7.1×10^−7^). There are minor differences in the ‘VEGF signaling’ (FDR_Younger_=1.1×10^−8^ versus FDR_Aged_=6.5×10^−7^), but the interacting partners are distinct. Thus, Neuropilin 1 (*NRP1*) and Angiopoietin 1 (*ANGPT1*) appear enriched in the Younger Group, whereas Neuropilin 2 (*NRP2*), Angiopoietin 2 (*ANGPT2*), Angiopoietin Like 4 (*ANGPTL4*), and Paxilin (*PXN*) are enriched in the Aged Group.

Although IL-4 is a potent mitogen on ECs (Kato et al., 2003), its impact cannot be evaluated directly because both positive and negative regulations appear compromised (see Fig. 2A). However, by comparing the evolutions of IFN-γ (Fig. 2I), TNF-α (Fig. 2E), and the adhesion molecules ICAM-1 and VCAM-1 (see ‘shear stress, cell adhesion, and extracellular matrix’ section), against observations gathered by Colotta et al. (1992) and Skaria et al. (2016), we have to conclude that in the Younger Group endothelial cells must be in proliferating state because IL-4 would have to be necessarily elevated.

Supporting that ECs are proliferating in the Younger Group, we have not detected transcription of markers of endothelial quiescence such as IL-33 (Sundlisaeter et al., 2012), nor S1PR5 (van Hooren et al., 2014), nor the gatekeeper FOXO1 (Wilhelm et al., 2016). Instead, IL-33 dysregulation was detected in the Aged Group. Moreover, transcripts of NOTCH and VEGFR3 both peaked in the Aged Group (see Fig. 2F). NOTCH receptor activation, induces the synthesis of VEGFR3/FLT4, which contributes to the quiescent stage of ‘stalk’ endothelial cells (Heinolainen et al., 2017).

### Shear stress, cell adhesion, and extracellular matrix

**Fig. 3.**
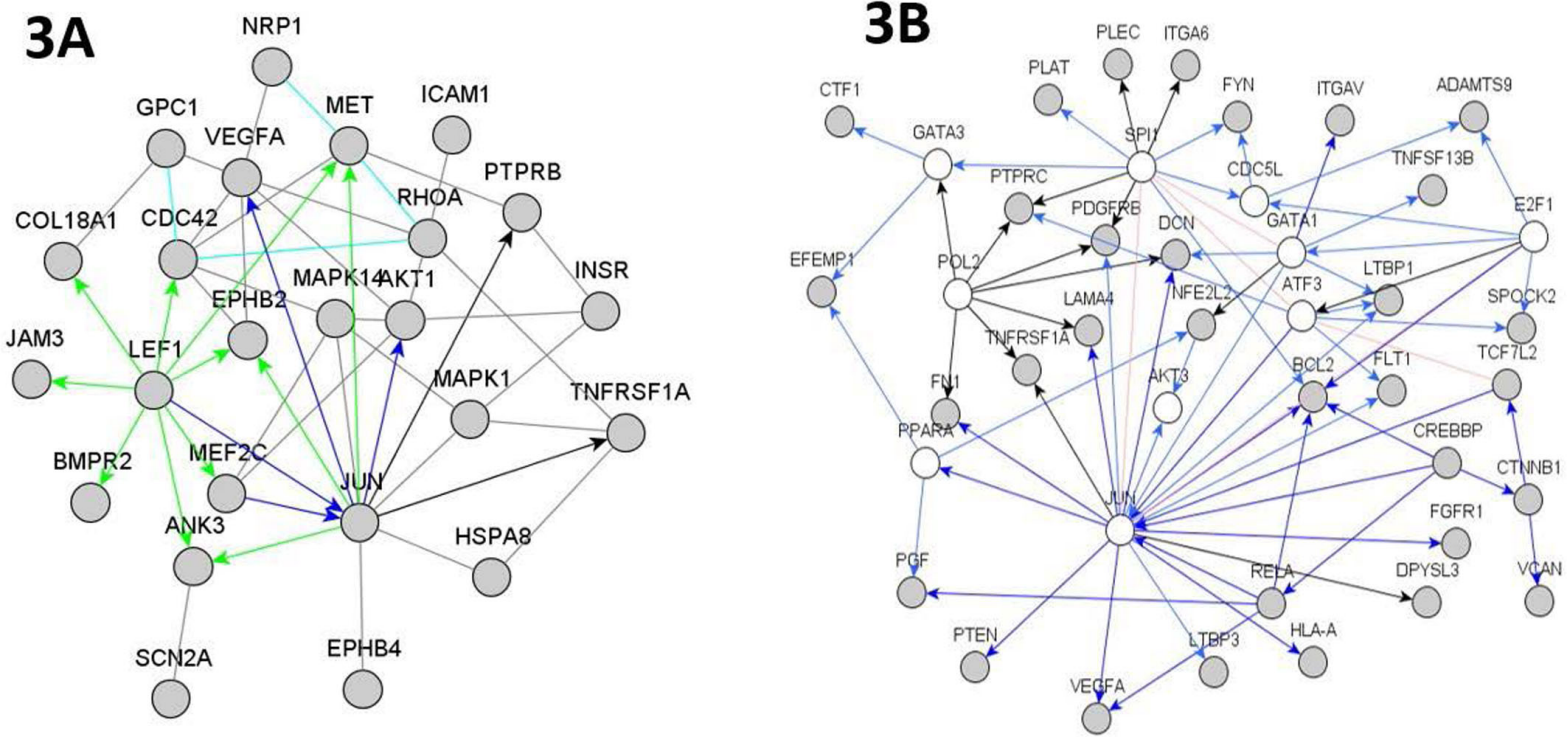
**Core networks arising from the analysis of the interaction between the cell adhesion molecules (CAMs) and the extracellular matrix (ECM).** (A) Younger Group; (B) Aged Group. Nodes colored in grey represent the significant differential molecules, while those colored in white are connecting nodes added to avoid that significant nodes remain isolated. Blue arrows represent well established genetic relationships; green arrows represent predicted genetic relationships; black arrows account for transcription factors. Lines colored in grey represent protein–protein interactions.

The analysis of molecules that make up the extracellular matrix, together with those that interact to (or regulate) them, such as the so-called cellular adhesion molecules (CAMs) also provided direct and indirect valuable information (see Fig. 3A and B).

A differential feature is given by the ‘response to shear stress’ (FDR_Younger_=1.3×10^−11^ versus FDR_Aged_=4.5×10^−4^). In the Younger Group, this response involved the following genes: *GPC1*, *RHOA*, *JUN*, *BMPR2*, *MAPK14*, *ICAM1*, *PRKAA1*, *AKT1*, *TNFRSF1A*, *TXN2*, *NOS3*, *MEF2C*, *VCAM1*, *VEGFA*, whereas in the Aged Group it comprised of *PECAM1*, *RELA*, *BCL2*, *ITGAV*, *BMPR1B*, *NFE2L2*, *PLAT*, *CTNNB1*, *TNFRSF1A*, *VEGFA*. In contrast, the ‘extracellular matrix organization’ and ‘proteoglycans synthesis’ prevailed in the Aged Group: *LAMA4*, *ITGA6*, *ITGAV*, *DCN*, *EFEMP1*, *ADAM17*, *ADAMTS9*, *FN1*, *PECAM1*, *VCAN*, *VWF*, *CTF1*, *PLAT*, *PDGFC*, *VEGFA*, *TNFSP13B*, *TGM2*, *PGF*, *PLXND1*, *SPOCK2*, *CLEC7A*, *LTBP1*, *LTBP3*, *PLEC*, *WNT5A*, *IGFBP3* (FDR_Aged_=4.5×10^−4^).

Both the Younger Group and the Aged Group showed a dysregulated transcription of genes corresponding to CAMs. However, the genes involved in each group are substantially different: *Col18A1*, *PSEN1*, *ICAM1*, *VCAM1*, *JAM3* (FDR_Younger_= 4.4×10^−2^) versus *HLA-A*, *PECAM1*, *ITGAV*, *ITGA6*, *CD40*, *PTPRC*, *VCAN* (FDR_Aged_=8.5×10^−3^). Importantly, endothelial cells in the Younger Group showed not only ‘homotypic cell adhesion’, but also ‘heterophilic cell adhesion’ [*SELP*, *ICAM1*, *VCAM1* (FDR_Younger_=3.5×10^−3^)]. In fact, the Younger Group showed ‘leukocyte adhesion to the blood vessel wall’ [*IL-12B*, *HLA-A*, *ITGB1*, *ITGB2*, *ETS1*, *PSEN1*, *CASP3*, *LGALS8*, *HMGB1*, *HSP90AA1*, *ROCK1* (FDR_Younger_=2×10^−11^)], mainly macrophages, monocytes, and lymphocytes (FDR_Younger_=10^−7^ to 10^−9^).

The dysregulation of sphyngosine-1P receptor signaling, IL-4, and HSPAA8 in the Younger Group could reflect an anti-inflammatory response to balance the inflammatory environment due to Thromboxane A2 and other cytokines in this group (see Fig. 2E). The dysregulation of VCAM-1 and ICAM-1 in the Younger Group is consistent with our results about RAGE activation, NOD-like signaling, HMGB1 occurrence, TLRs activation (‘RAGE’ section), and the effects of Reelin on LRP8 (‘Lipidic alterations’ section), as well as with other reported data (Guo et al., 2019; Yousef et al., 2019; Zhao et al., 2020).

JAM-3 and Selectin-P also appeared associated to the increments of VCAM-1 and ICAM-1 in the Younger Group. Noticeably, PECAM-1, which is required for trans-endothelial migration of leukocytes, appeared dysregulated in the Aged Group, but not in the Younger Group. Hence, the heterophilic cell adhesion detected in the Younger Group could reflect the normal mechanism of immune surveillance, without leukocyte transmigration, but probably after having completed the previous steps of tethering, rolling down, and crawling (Marchetti and Engelhardt, 2020). In fact, it has been reported that immune response is compromised in aging, as well as in other neurovascular pathologies (Guo et al., 2019). A final conclusion in this regard, will require a direct experimental verification.

### Artery and vein formation at the younger-aging transition

‘Artery morphogenesis’ prevailed in the Aged Group (FDR_Younger_=2×10^−3^ versus FDR_Aged_=2.7×10^−8^), whereas ‘Venous morphogenesis’ appeared dysregulated in both groups (FDR_Younger_=1.4×10^−3^, FDR_Aged_=1.5×10^−2^).

Arterial morphogenesis is in line with the fact that ‘VSMC proliferation’ peaks in the Aged Group (see Fig. 2D). However, while proliferation of VSMCs was associated in the Younger Group to significant ‘VSMC chemotaxis’ and ‘VSMC migration’, this did not occur in the Aged Group. These differences open the question about the functionality of the artery morphogenesis detected. Data seem to pinpoint that artery morphogenesis in the Aged Group could involve mainly a hypertrophic process of the muscular layer surrounding the arteries and arterioles. Moreover, the artery morphogenesis in the Aged Group is supported also by the detection of a dysregulated constellation of characteristic genes (*HES4*, *JAG2*, *NOTCH1/2*, *EFNA1*, *EFNAB2*, *SOX6*, *VCAM1*, *BGN*, *COL1A2*, *COL5A*, *FN1*, *LAMA4*, *ITGAV*, *VWF*, *ECE1*, *EDN1*, *BMP4*, *LTBP1*; Cui et al., 2015).

Interestingly, the Younger Group appeared enriched in the transcripts of genes *HOXA7*, *HOXC6*, and *HOXD3*, whereas enrichment in *HOXA10*, *HOXD8*, *HOXB1*, *HOXC11* occurred in the Aged Group. In the Younger Group, all the HOX factors detected are clearly associated to pro-angiogenic effects (Chung et al., 2009; Toshner et al., 2014). In the Aged Group, HOXC11 inhibits vascular remodeling. On the contrary, HOXB1 is abundantly present in VSMCs during remodeling (Pruett et al., 2012). Importantly, HOXA10 and HOXD8 in the Aged Group are responsive to a low shear stress rate. As we discuss below, this phenomenon can be caused by an increased tortuosity of the blood vessels, as well as to the occurrence of atheroma (Souilhol et al., 2020).

### Tortuosity, shear stress, and vascular inflammation

**Fig. 4.**
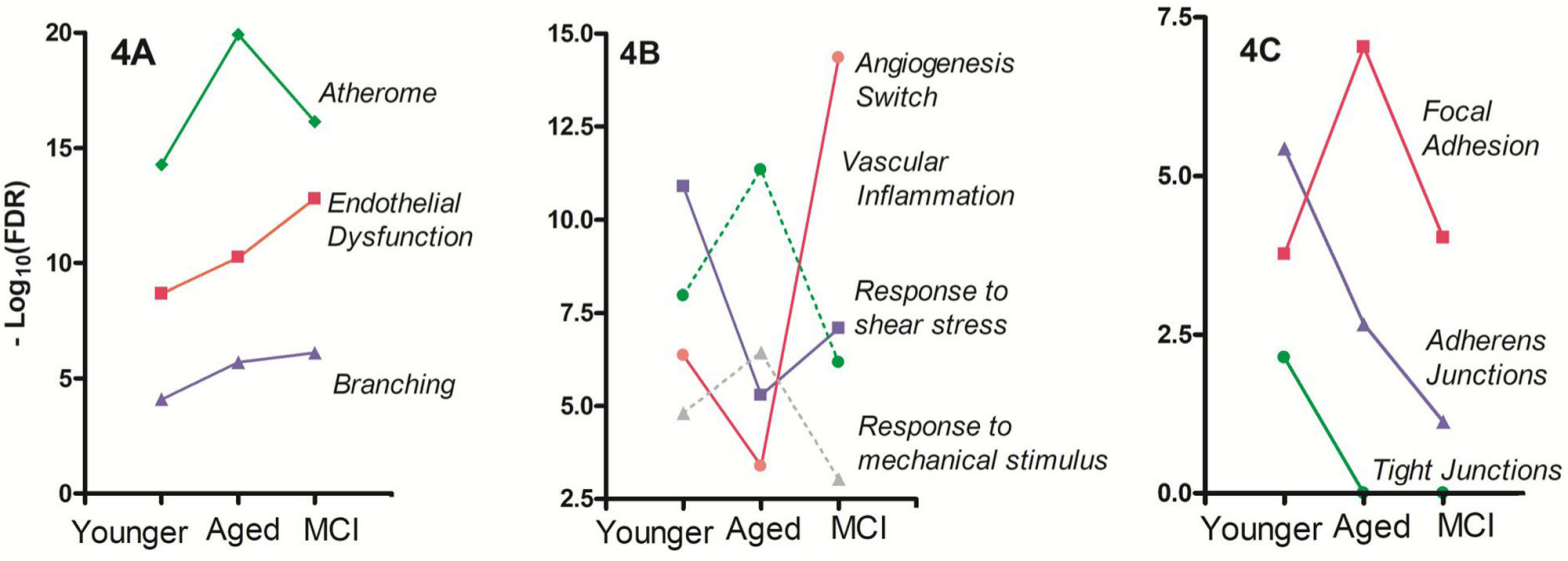
**Evolution of some indicators of endothelial cell physiology across the three conditions analyzed (Younger Group), Aged Group, and MCI Group).** (A) Parallelism between blood vessel branching, the degree of endothelial dysfunction and degree of atherosclerosis; (B) Evolution of vascular inflammation (dashed green line), a measure of ‘angiogenic switch’ (red line), cellular response to shear stress (solid violet line), and cellular response to mechanical stimulus (dashed grey line); (C) Evolution of structures involved in the adhesion of the endothelial cells (adherens junctions, tight junctions, focal adhesion). Data corresponding to GAP Junctions are not shown, but they overlap with the curve of tight junctions.

Because of their enrichment in *COL4A3*, *COL4A4*, *TGFBR2*, *TGFB3*, and *SMAD3*, the blood vessels in the Aged Group could have a significant incidence of ‘tortuosity’ (FDR_Aged_=2.9×10^−3^). Tortuosity affects not only arteries and arterioles, but also veins and capillaries. The excess of tortuosity, through its effects on the local variation of the shear stress (Han, 2012), is a cause of endothelial dysfunction (Cousins et al., 2018; Veluchamy et al., 2019; Hachana et al., 2020). In turn, endothelial dysfunction is frequent in diseases associated to cognitive limitations such as diabetes (Hadi and Suwaidi, 2007) and Alzheimer's disease (Kelleher and Soiza, 2013). In Fig. 4A how the ‘endothelial cell dysfunction’ increased together with the branching of the blood vessels is depicted. That branching can be taken as a surrogate measure of the tortuosity factor in our analysis.

Importantly, vascular cells respond with distinct programs of gene expression according to the local shear stress received (Han, 2012; Krüger-Genge et al., 2019). In this direction, a clear increment in the significance of ‘atherosclerosis’ is observed in the Aged Group (see Fig. 4A). Both the Younger Group and the Aged Group exhibited ‘vascular inflammation’ (FDR_Younger_=1×10^−8^, FDR_Aged_=1.2×10^−10^) (see Fig. 4B). As atherogenic process begins as an infiltrate of monocytes in the tunica media of the arteries, it will mainly affect the smooth muscle cells. Consistently, the endothelial cells in the Aged Group showed an increased significance of ‘response to mechanical stimulus’ (see Fig. 4B).

Noticeably, local variations in the shear stress can explain the occurrence of local hypoxic micro-regions along the brain vasculature. This could be the case of the ‘cellular response to hypoxia’ observed in both ‘healthy’ groups studied (FDR_Younger_=1.2×10^−10^, FDR_Aged_=2.7×10^−10^). However, these responses involved distinct genes in each case (Younger Group: *EPAS1*, *ADORA1*, *CAMK2D*, *ICAM1*, *VCAM1*, *PRKAA1*, *ETS1*, *AKT1*, *PRKCQ*, *PPARA*, *TXN2*, *CX3CL1*, *EPAS1*, *CXCL12*, *VEGFA* versus Aged Group: *MYC*, *FOXO3*, *FLT1*, *HIF1A*, *FOSL2*, *BCL2*, *PPARA*, *PTEN*, *ECE1*, *CREBBP*, *PGF*, *TGFB1*, *CFLAR*, *TGFBR3*, *NFE2L2*, *PLAT*, *KDR*, *ADAM17*, *ENG*, *VEGFA*). Also, distinct target genes were transcribed by the factors HIF1α and HIF2α in each case [Younger Group: *JUN*, *GATA2*, *MAPK1*, *ETS1*, *AKT1*, *CAMK2*, *INSR*, *NOS3*, *VEGFA*, *CXCL12*, *EPAS1* (FDR_Younger_=1.3×10^−3^) versus Aged Group: *SP1*, *HIF1A*, *GATA2*, *MCL1*, *CREBBP*, *ABCB1*, *ENG*, *VEGFA*, *FLT1*, *EFNA* (FDR_Aged_=2.9×10^−4^)].

Consistently with the occurrence of hypoxia, in both groups we have also detected a dysregulated generation of reactive oxygen species (ROS) (FDR_Younger_=1.3×10^−5^, FDR_Aged_=6.5×10^−5^) as well as in the reactive nitrogen species (RNS) (FDR_Younger_=8.0×10^−5^, FDR_Aged_=7.37×10^−6^). However, there was an asymmetry in the ‘response to oxidative stress’ (FDR_Younger_=1.7×10^−6^ versus FDR_Aged_=7.5×10^−10^). The response to oxidative stress in the Younger Group comprised the genes *FOXO3*, *MET*, *JUN*, *PSEN1*, *PDGFD*, *PRKAA1*, *ETS1*, *AKT1*, *TXNRD2*, *TXN2*, *NOS3*, *EPAS1*, *SOD2*, whereas in the Aged Group it was given by *FOXO3*, *EPOR*, *SP1*, *HIF1A*, *PDGFRB*, *AXL*, *MCL1*, *RELA*, *BCL2*, *PRDX2*, *KLF4*, *ITGAV*, *CFLAR*, *EGFR*, *NFE2L2*, *TLR4*, *AKR1C3*, *PXN*, *CTNNB1*, *FYN*, *HSPA1B*, *NOS3*, *SOD2*.

The response to oxidative stress in the Aged Group has some interesting features: First, the transcription factor NEF2L2 (nuclear factor, erythroid 2 like 2) is the central regulator for the transcription of genes related with ROS detoxifying enzymes (Nguyen et al., 2009). In addition, the transcription factor KLF4 (Krüppel-Like4) is induced in response to hypoxia or glucose deprivation. KLF4 inhibits the proliferation of VSMCs (Yoshida et al., 2014), while it exerts a protective, anti-apoptotic, anti-inflammatory effect on the endothelium (Yang et al., 2018). Interestingly, in the Aged Group the dehydrogenase AKR1C3 (prostaglandin F synthase) is involved in the metabolization of estrogens, androgens and prostanoids. These molecules can regulate the oxytocin receptors, which, when activated in the blood vessels, can inhibit the attachment of monocytes to oxidized-LDL receptors during atheroma formation (Penning et al., 2000; Liu et al., 2017). Importantly, oxytocin can also be transported through the BBB by the receptors for the advanced glycation end-products (RAGE), participating in the generation of ROS (Wang et al., 2019; Yamamoto et al., 2019).

### Inflammatory context

Although the item ‘inflammation’ peaks in the Aged Group, it is evident that the Younger Group exhibits a sub-inflammatory response, which can be characterized as a phenotype of ‘endothelial cell activation’ (Liao, 2013). In the Aged Group, functionalities as the ‘production of cytokines’, ‘synthesis of eicosanoids’, and ‘response to prostanoids’ are maximized (see Fig. 2D), whereas that of the ‘response to thromboxane’ is minimized (see Fig. 2E). Both Younger and Aged Groups show a dysregulated leukotriene C4 synthase (LTC4S), a transferase that consumes glutathione to incorporate cysteine to the production of the leukotriene molecule. Note that prostaglandins, thromboxane A, and leukotrienes, all derive from arachidonic acid. However, thromboxane is synthesized mainly by activated platelets, whereas prostanoid/prostaglandins can be synthesized by the ECs, and leukotrienes by leukocytes (Hanna and Hafez, 2018).

Both the Younger Group and the Aged Group exhibited significant ‘arachidonic acid signaling’ (FDR_Younger_=7,11×10^−3^, FDR_Aged_=2.4×10^−2^), but ‘thromboxane A signaling’ prevailed in the Younger Group (FDR_Younger_=3.3×10^−7^ versus FDR_Aged_=4.46×10^−2^). Conversely, the ‘production of prostanoids and prostaglandins’, particularly the prostaglandin E2 (PGE2), prevailed in the Aged Group (FDR_Aged_=3.4×10^−3^), whereas it was not significant in the Younger Group (FDR_Younger_=0.17). Note that thromboxane A is a pro-inflammatory, procoagulant, and vasoconstrictor factor, whereas PGE2 is anti-inflammatory.

According to their different mechanisms of induction and their multiple crossed regulations, distinct inflammatory molecules showed distinct temporal profiles. For instance, IL-1 signaling increased across the three conditions tested (see Fig. 2B), whereas Interferon-γ (IFNG) was practically constant up to the Aged Group (see Fig. 2I). Instead, other interferons (IFN-α, IFN-β) increased from the Younger Group to the Aged Group, where the high plateau persisted in the MCI group (see Fig. 2F). This last pattern can be understood as a reactive response to the occurring inflammation mediated by IL-1, since IFN-β is a potent anti-inflammatory molecule (Kuo et al., 2016).

The ‘TNF-α signaling’ in the Younger Group showed a slight diminution in the Aged Group, but increased further in the MCI stage (see Fig. 2E). The transitory diminution of the TNF-α could be explained in the Aged Group by the exacerbated ‘response to TGF-β’ (Welser et al., 2010; Welser-Alves and Milner, 2013) (see Table S1).

Interestingly, the evolution of TGF-β is paralleled by peaking of the latent TGF-β (LTGFB) as well as by the loss of negative regulation of TGF-β. The activation of LTGFB implies the mobilization of TGF-β stored in the extracellular matrix (ECM), which is remodeling because of the occurring peak of inflammation (see Fig. 2D).

In fact, differential categories in the Aged Group were: the ‘ECM organization’ (FDR_Aged_ =4.5×10^−4^), ‘ensemble of ECM genes’ (FDR_Aged_=8.4×10^−3^), and ‘proteoglycans’ (FDR_Aged_=2.2×10^−2^). The ‘ensemble of ECM genes’ was associated to *VWF*, *FN1*, *LAMA4*, *SPOCK2*, *DCN*, *EFEMP1*, *IGFBP3*, *VCAN*, *LTBP1*, *LTBP3*, *WNT5A*, *CLEC7A*, *PLXND1*, *PGF*, *TGM2*, *ADAMTS9*, *TNFSF13B*, *PLAT*, *CTF1*, *ADAM17*, *PDGFC*, *VEGFA*, while the ‘ECM organization’ associated with the genes *FN1*, *PECAM1*, *LAMA4*, *ITGA6*, *DCN*, *ITGAV*, *EFEMP1*, *ADAMTS9*, *VCAN*, *ADAM17*, *LTBP1*, *LTBP3*, *PLEC*. Proteoglycans comprised *VWF*, *FN1*, *LAMA4*, *EFEMP1*, *IGFBP3*, *LTBP1*, *LTBP3*, *ITGA6*, *ITGAV*, *DCN*, *VCAN*. Also peaking in the Aged Group were the transcripts of the interleukins IL-8, IL-12, IL-13. Instead, ‘IL-6 signaling’ appeared as non-significant in the Younger Group but it raised and persisted at high levels of significance in the Aged and MCI Groups (see Fig. 2F).

## Brain insulin-resistance

The Younger Group and the Aged Group showed minor differences in the ‘insulin receptor signaling’ (FDR_Younger_=4.3×10^−3^, FDR_Aged_=1.2×10^−2^), but differ about the ‘cellular response to insulin’ (FDR_Younger_=2.5×10^−4^ versus FDR_Aged_=4.4×10^−2^) and in the ‘sensitivity to insulin’ (FDR_Younger_=3.7×10^−6^ versus FDR_Aged_=8.1×10^−3^). The ‘insulin receptor signaling’ in the Younger Group involved the genes *MET*, *KITLG*, *PRKAA1*, ***INSR***, *AKT1*, *MAPK1,* whereas in the Aged Group it involved the genes *VWF, FN1*, *PDGFRB*, *CAMK2B*, *CSF2RB*, *FGFR1*, *IL17RD*, *FYN*. The ‘insulin sensitivity’ in the Younger Group was given by the dysregulation of the genes *PPARGC1B*, *PPARA*, *AKT1*, *NOS3*, *INSR*, *PRKAA1*, *PRCKQ*, *TNFRSF1A,* whereas in the Aged Group it appeared linked to the genes *FOSL2*, *THRA*, *STAT3*, *PPARA*, *PTEN*, *CTF1*, *CREBBP*, and *NFE2L2*.

Concerning the receptor MET in the Younger Group, its activation by hepatocyte growth factor (HGF) triggers a positive crosstalk with INSR (insulin substrate receptor). As result, MET activation exerts an anti-inflammatory effect by diminishing the secretion of TNF-α, IL-6, and MCP-1 (Oliveira et al., 2018). However, while INSR was significant in the Younger Group, it does not appear as significant in the Aged Group. In the Aged, it was the PDGF receptor (PDFGR) that appeared as significant instead. PDGFR could be responsible for a diminished activity of PI3 K, AKT, and NOS3 in this group (Myers et al., 1995; Talbot et al., 2012). Hence, the weak activation of INSR by PDGFR could explain both the ROS diminution as well as the increased NRS production observed in the Aged Group (see ‘Tortuosity, shear stress and vascular inflammation’ section). Interestingly, in the Younger Group the thyroid hormone receptor beta (THRB) predominates, whereas its alpha form (THRA) appears in the Aged Group. While THRA activation develops angiogenic effects (Talhada et al., 2019), the THRB activation leads to anti-angiogenic effects (Kim et al., 2014).

In brief, the sensitivity to insulin reaches a minimum in the Aged Group and is further maintained at low level in the MCI stage (see Fig. 2G). This kind of response can be understood as a phenomenon of brain resistance to insulin (Lannert and Hoyer, 1998; Talbot et al., 2012; Matioli and Nitrini, 2015). The brain insulin resistance can be caused by the low transport of insulin through the BBB, the downregulation of insulin receptor, by the low binding of insulin to its receptors, failure in the cascade signaling down-stream of the insulin receptor, and/or exacerbated insulin clearance by the insulin degrading enzyme (IDE) (Arnold et al., 2018).

Peripheral insulin resistance is frequent in advanced cases of type II diabetes, and has been associated to impaired cognition and/or mood disorders. However, similar symptoms can also appear in brain insulin resistance, even without any evidence of diabetes. Insulin resistance in the brain is considered as a risk factor for Alzheimer’s dementia, but cognitive limitations in type II diabetes are not accompanied by senile plaques (Aβ peptide), nor neurofibrillary tangles (Tau protein). Interestingly, cerebral endothelial cells coming from an experimental model of diabetic mice showed very few common dysregulated genes respect to the observed in the ‘aged’ mice. However, one of the main shared features was the diminution of the immune response (Guo et al., 2019).

### RAGE

The signaling through the RAGE receptor prevailed in the Younger Group (FDR_Younger_=2.6×10^−7^ versus FDR_Aged_=7.1×10^−3^). In the Younger Group, this feature is supported by the differential transcription of *ICAM1*, *VCAM1*, *VEGFA*, *CDC42*, *JUN*, *MAPK14*, *MAPK*, which is compatible with the indicators of the ‘activation state’ of these endothelial cells (Harja et al., 2008). Importantly, note that RAGE receptor not only binds advanced end glycation products (AGEs), but can also recognize other ligands carrying sequences of the pathogen-associated molecular patterns (PAMPs) and host-derived damage-associated molecular patterns (DAMPs) (Teissier and Boulanger, 2019).

In fact, we detected a dysregulated transcription of the DAMP-protein HMGB1 (high mobility group box 1 protein) in the Younger Group. The protein HMGB1 is capable to translocating to distinct localizations (nucleus, cytoplasm, extracellular), to perform different functions. HMGB1 can act as ligand and inducer of RAGE, but depending on the context, it can develop angiogenic effects and/or inflammatory effects, which include exacerbated induction of ICAM-1, VCAM-1, SEL-P (Luo et al., 2013). When RAGE works coupled to the oxidase NOD, it prompts ROS generation (Volpe et al., 2018). Consistently, we also detected a dysregulated ‘NOD-like signaling’ in the Younger Group (FDR_Younger_=7.25×10^−3^). Again, the transcription of ICAM-1 and VCAM-1 indicates that NFKB is operating in the Younger Group, and that endothelial cells are thus ‘activated’ (Derk et al., 2018). In agreement with the fact that RAGE activation is in crosstalk with toll-like receptors (TLRs) (Prantner et al., 2020), we detected ‘TLR4 and TLR9 signaling’ in the Younger Group (FDR=1.9×10^−3^).

However, RAGE has 19 isoforms. Most of them localize at the plasma membrane, but two are extra-cellular [the so-called ‘soluble’ form (s)RAGE, and the ‘endogenous secretory’ (es)RAGE]. While (s)RAGE is generated by shedding from the membrane-bound RAGE, (es)RAGE is synthesized by alternative splicing (Egaña-Gorroño et al., 2020). The differentiation between the RAGE forms is important because the activation of the membrane-bound RAGEs usually leads to oxidative stress and inflammation, particularly when lipopeptides (derived from LPS) or the peptide Aβ (derived from APP protein) are bound (Sagare et al., 2013; Zhao et al., 2015). On the contrary, the (es)/(s)RAGE forms function as decoy receptors. In fact, a sharp diminution of the (s)RAGE in patients with Alzheimer's disease has been reported (Emanuele et al., 2005), while RAGE activation in macrophages participates in the genesis of atherosclerosis (Díaz-Casasola and Luna-Pichardo, 2016). Multimodal RAGE inhibitors are being developed to mitigate these effects (Deane et al., 2012; see also https://clinicaltrials.gov/ct2/show/NCT03980730).

Herein, we cannot discern whether the enhanced signaling through the RAGE receptor in the Younger Group is associated to a plasma membrane form, to a soluble form, or to a mixture of both. This last situation seems compatible with the low-grade inflammatory context detected in the Younger Group (‘Shear stress, cell adhesion and extracellular matrix’ section and Fig. 4A,B), but further experimental verification is required.

### Lipidic alterations

A remarkable finding is the occurrence of a lipidic-inflammatory disorder of the hippocampal vasculature, compatible with some degree of ‘atherosclerosis’ in the Younger Group (*n*=28 genes, FDR_Younger_=5.4×10^−15^). This feature peaks in the Aged Group (*n*=48 genes, FDR_Aged_=1.2×10^−20^) and persists in the MCI Group (*n*=22 genes, FDR_MCI_=7×10^−17^). Moreover, ‘atherosclerosis’ evolves in close parallelism to other related items, such as ‘vascular inflammation’ and ‘response to mechanical stimulus’ (see Fig. 4A,B).

Atherogenesis is a very complex, multi-step disorder (Emini Veseli et al., 2017; Kobiyama and Ley, 2018). In this regard, some common functionalities of the genes participating in atherogenesis across the groups is evidenced. Such is the case of several cell adhesion molecules (*SELP*, *ICAM1*, *VCAM1*, *CD14*, *PECAM1*, *CD44*, *SELE*), which drive the cellular infiltration of the blood vessel wall, following the chemo-attraction provided by several chemical gradients (*VEGFA*, *PDGFD*, *FGF2*, *ANGPT1*, *ANGPT2*, *WNT5A*, *CXCL12*, *FGF2*, *IL18*). This infiltration is the consequence of an inflammatory environment generated by cytokines, as well as by the activation and/or transactivation of integrins (*ITGAV*, *ITGB3*) and TLRs (*TLR3*, *TLR4*, *TLR9*).

The balance of lipids in the brain has upmost relevance to maintain the neurophysiological activities, and the protein ApoE has a crucial role connecting the different players within the neurovascular unity (Zhang and Liu, 2015; Saint-Pol and Gosselet, 2019). However, through their interactions with the LRP receptors, the ApoE-containing lipoproteins not only regulate the lipids (Actis-Dato and Chiabrando, 2018), but strongly influence APP processing and Aβ transport (Sagare et al., 2013; Jiang et al., 2014). The peptide Aβ – besides its direct neurotoxic effects – also negatively affects the insulin signaling in the brain (Gali et al., 2019).

We have detected that the Younger Group is featured by the receptor LRP8, whereas LRP1 predominates in the Aged Group. Although both receptors recognize ApoE-containing lipoproteins, they are not equivalent. Note that the clearance of the peptide Aβ will depend on the type of ApoE variant present. Hence, while LRP8 has the same affinity for all the ApoE isoforms (ϵ2, ϵ3, ϵ4), LRP1 has different affinities (ϵ3>ϵ2>>ϵ4) (Conejero-Goldberg et al., 2014). In fact, the occurrence of ApoE(ϵ4) isoform, whether in homozygous or heterozygous form, is a highly predictive risk factor of Alzheimer’s disease (Montagne et al., 2020). Moreover, the production of nitric oxide in endothelial cells is differentially affected by distinct isoforms of ApoE (Sacre et al., 2003).

LRP8 in the Younger Group could offer some advantages. It improves the anti-coagulant balance by contributing to neutralize the side-effects of prothrombin on the barrier properties of the endothelial cells (Sinha et al., 2016). Likewise, the activation of LRP8 in the platelets contributes to inhibit their aggregation (Riddell et al., 1999). Moreover, LRP8 prevents VSMCs from acquiring a senescent, secretory, fibrotic phenotype (Komaravolu et al., 2019). Interestingly, LRP8 acts as receptor for the protein Reelin. The binding of Reelin activates the PI3 K/AKT route (Dlugosz and Nimpf, 2018), while it also enhances ICAM-1 and VCAM-1 expression, thus increasing the adhesion of leukocytes to the endothelial cells (Ding et al., 2016).

Both LRP8 and LRP1 receptors are localized at the abluminal side of endothelial cells, but LRP1recognizes more than 40 ligands (Sagare et al., 2013). A ‘soluble’, extracellular fragment of LRP8 [(s)LRP8] can be generated by shedding the ectodomain from the mature receptor, while the auto-activation of the enzyme γ-secretase releases its intracellular domain (LRP8_ICD_) to the cytoplasm (May et al., 2003). It has been reported that (s)LRP8 increases with the age of individuals (Liu et al., 2009a), while the translocation of LRP8_ICD_ to the nucleus, turns it into a co-regulator of PPAR-γ dependent target genes (Mao et al., 2017).

The receptor LRP1 could be involved in some of the negative effects that we detected in the Aged Group. Thus, the dysregulation of BMPER and its interaction with LRP1 (Pi et al., 2012) is in line with the diminished angiogenesis observed (see Fig. 2A). Also, the detected increment in the resistance to insulin (‘Brain insulin-resistance’ section) seems be in line with the observations of Mao et al. (2017) because in the Aged Group the *PPARA* gene – but not the *PPARG* gene – appeared dysregulated. Instead, the genes *PPARG* and *PPARGC1B* genes appeared dysregulated in the Younger Group.

We also detected signatures compatible with ‘hypercholesterolemia’ in the analyzed series (FDR_Younger_=5.9×10^−6^, FDR_AGED_=1.2×10^−7^, FDR_MCI_=4.0×10^−17^). The Aged Group showed a dysregulated transcription of the enzyme HMGCR (which speeds up the synthesis of cholesterol), the ABCB1 ATPase-pump (it exports phospholipids, drugs, and peptide Aβ), and the receptor SCARB1 (which clears cholesterol from the lipoproteins). These effects are also potentiated by the enhanced the transcription of the genes *SREBF* and *CREBBP* (Chandra et al., 2008). In the MCI Group, the dysregulation of the nuclear factor NR4A1, represses the induction of the genes *HMGCR*, *LDLR*, *SREBP1*, while it tends to re-establish the carbohydrate balance (Zhang et al., 2018).

### MCI specific changes

Only two out of the ten patterns that we have detected can be attributed to specific changes in the MCI (see Fig. 2H and I). While functionalities grouped in Fig. 2H are upregulated (response to corticotrophin-releasing hormone, lactosylceramide signaling, and IL-15/18/21/23 signaling), those grouped in Fig. 2I are downregulated (positive regulations of IL-2, TGF-β, IFN-γ, nitric oxide, and chemokine production).

Concerning the interleukins up-regulated in the MCI Group, IL-15 is produced by endothelial cells after their stimulation by TNF-α and IFN-γ, leading to their chronic activation (Liu et al., 2009b). The stimulation of VSMCs by prostaglandin PGE2 also induces IL-15. In this case IL-15 inhibits the proliferation of VSMCs, but not their migration (Iwasaki et al., 2007). Because IL-15 binds to a receptor complex made up by IL15RB and IL2RB, IL-15 is in competition with IL-2. This point is important, because the level of T-cells results from the interplay between IL-2 and IL-15. On the other hand, IL-18 arises from a paracrine relationship between its production by macrophages and T-cells in the atheroma plaques, and its targets, i.e. ECs, VSMCs, and monocytes that express the corresponding receptor. As result, the endothelial cells acquire a marked inflammatory-adhesive phenotype (Gerdes et al., 2002; Tielemans et al., 2019). Moreover, inflammation associated to IL-18 compromises some cognitive abilities as learning and memory (Wu et al., 2016).

In full agreement with Gerdes et al. (2002) and Tielemans et al. (2019), our observations also state the very poor significance of tight junctions in MCI (see Fig. 4C), as well as the detected differential transcription of ICAM-1, VCAM-1, SELE, and CDH5/VE-cadherin (FDR_MCI_=7.3×10^−3^). Note that there is no conflict between the scarcity of adherens junctions in MCI (see Fig. 4C) and the expression of CDH5 in the same group. In fact, CDH5 can be synthesized and accumulated as distinct phosphorylation species, and/or be part of a complex CDH5-β-catenin-IQGAP without being incorporated into the adherens junctions (Guebel et al., 2012).

The dysregulation of lactosylceramide was evidenced by the upregulation of the gene codifying the enzyme B4GALT6 (FDR=7.55×10^−3^). VEGFA and FGF2, both lead to the synthesis of lactosylceramide, which enhances the angiogenic effect of its inducers (Kolmakova et al., 2009). In the VSMCs, lactosylceramide enhances their proliferation and migration (Mu et al., 2009), while in macrophages and neutrophils it favors their transmigration (Mayo et al., 2014).

The differential response to corticotrophin-releasing hormone (CRH) in the MCI Group was evidenced by dysregulation of the nuclear receptor genes *NR4A1/Nur77*, *NR4A2/NURR1*, and *NR4A3/Nor1* (FDR_MCI_=1.6×10^−6^). Hippocampal vessels express the receptors CRHR1 and CRHR2 (Deussing et al., 2007), so that their stimulation by CRH leads to the activation of the family of nuclear receptors NR4A (Liu et al., 2003). Once activated, NR4A1 and NR4A2 participate in the response of endothelial cells to injuries (Theoharides and Kostantinidou, 2007; Safe et al., 2016). Moreover, NA4A3 has positive effects on the proliferation of VSMCs, while it also favors the adhesion of monocytes by inducing VCAM-1 and ICAM-1 (Deussing et al., 2007).

The arteriogenesis that was observed in the MCI Group (FDR_MCI_=7.6×10^−6^) could be explained by the convergent effects of NR4A3 and lactosylceramide on the VSMCs, together with the effects of NR4A1 on the endothelial cells. Moreover, arteriogenesis appeared associated to an intense proliferation of VSMCs (FDR_MCI_=1.3×10^−8^). These features seem to result in the progression of the phenomena observed in the Aged Group (see Fig. 4A). The excessive vascular branching could produce negative consequences on the physiology of endothelial and smooth muscle cells due to the increased local variations in the shear stress rate (see ‘Tortuosity, shear stress, and extracellular matrix’ section).

### Checking the identification of genes

The performance of our computational pipeline was assessed by determining the intersection between the list of identified genes in each studied group with respect to the occurrence of these genes in some of the two data-sets taken as reference [X ∩ (Reference_1_ ∪ Reference_2_)]. In the Younger Group (*n*=65 genes), the accuracy was 98.5%, in the case of the Aged Group (*n*=147 genes), accuracy was 99.3%, while in the MCI Group (*n*=37 genes), accuracy resulted 97.3%. Hence, the inaccuracy in our approach resulted less than or equal to 2.7%. Even this maximum level of error does not seem to compromise the main biological inferences achieved, mainly because conclusions were not drawn on individual genes but from consistent networks.

### Concluding remarks

We designed a computational strategy to determine the state of vasculature in human hippocampus. The information was recovered from ‘global’ microarrays data gathered by Berchtold et al. (2008) and (2014). After appropriate filtering operations, the targeted information was isolated, and multiple aspects regulating the vasculature under healthy (Younger and Elderly Groups) and diseased conditions (MCI Group) were analyzed.

Our approach was chosen because specific information for the human hippocampal vasculature is lacking, despite the great advances in the transcriptomics of the cerebral vasculature (Zhang et al., 2014; Vanlandewijck et al., 2018; Munji et al., 2019; Guo et al., 2019; Yousef et al., 2019; Zhao et al., 2020). There is information from mouse hippocampal vasculature (Chen et al., 2020), but mouse and human are not completely equivalent models (Mestas and Hughes, 2004; Calabria and Shusta, 2008; Song et al., 2020; Zhang et al., 2020).

The methodology applied reveals many previously undetected features of angiogenesis in younger and aged individuals (Berchtold et al., 2008, 2014; Guebel and Torres, 2016). Our finding that endothelium in the (healthy) Younger Group is already in an ‘activated stage’ is of note, as well as the notions that this group exhibits active angiogenesis, associated to a significant homotypic and heterophilic cell adhesion (see sections from ‘Angiogenesis multidimensionality’ to ‘Shear stress, cell adhesion and extracellular matrix’). The (healthy) Aged Group showed an increment of several indicators (arterialization, vascular inflammation, vessel branching, brain insulin resistance, oxidative stress, atherosclerosis, focal adhesion) and diminution in others (sensitivity to shear stress, homotypic cell–cell adhesion structures) (see sections from ‘Artery and vein formation’ to ‘Lipidic alterations’). In the MCI Group, a small set of variables appeared specifically associated to this condition (see ‘MCI specific changes’ section), coexisting with others that suggest compensatory responses (see Figs 2D–E and 4B). Note that ‘healthy’ herein refers to donors whose brains were ‘neurologically healthy’. These individuals complied with well-defined criteria of inclusion/exclusion and with specific results arisen from cognitive evaluation (MMSE test), dementia evaluation (CDR test), and post-mortem brain Braak's staging (Berchtold et al., 2008, 2014).

The fact that some incipient features of vascular aging appeared in the Younger Group is not strange because these were middle-aged individuals. Hence, their biological and chronological ages were increasingly divergent (Fedintsev et al., 2017; Voisin et al., 2020; Shireby et al., 2020 preprint). Moreover, vascular alterations in ‘healthy’ younger individuals can be evidenced from other independent studies as well: (i) six-weeks-old rats were divided in two groups: one group received high-fat diet, whilst the other group received normal diet. The high-fat diet group showed high IgG brain vascular leakage beginning at the age of 3 months. The normal diet group acquired the same feature, but starting when 12 months old (their middle age) (Takechi et al., 2013); (ii) in an epidemiological study, a clear association between microvascular dysfunction and cognitive aspects was detected even after adjusting for illness-dependent covariates (Rensma et al., 2020); (iii) the indicator of inflammation (s)VCAM-1 increased significantly with age, but a group of ‘disease-free’ young mice (3 months old) showed a (s)VCAM-1 in blood as high as 70% of the value detected in the group of aged mice (19 months old) (Yousef et al., 2019); (iv) it was observed that brain vascular permeability increased with age in a DCE-MRI study. However, the k_transference_ for the tracer was not null in a group of healthy-young individuals (23 to 47 years old). The baseline was 66% of the value reached in an older group (Montagne et al., 2015). Similar features were observed in another study covering multiple brain regions (Nation et al., 2019).

We cannot draw conclusions about causal mechanisms because our results arise from a cross-sectional rather than from a longitudinal study. Our findings should be tested upon larger cohorts in a prospective mode, and/or subjected to verification by targeted experimental interventions.

In particular, it would be required to test whether the detected ‘arterialization’ in the Aged Group actually is or not a process of “capillary arterialization” (Rissanen et al., 2005; Mac Gabhann and Peirce, 2010; Gouin et al., 2018). In fact, anatomy of the hippocampal vasculature supports the occurrence of collateral circulation through anastomosis (Ogawa et al., 2018; Spallazzi et al., 2019). The ‘capillary arterialization’ alternative is attractive because it is in line with the proliferation of VSMCs, and the increased tortuosity detected (‘Artery and vein formation’ and ‘Tortuosity, shear stress, vascular inflammation’ section), as well as with the hierarchical mechanisms in the generation of vessels (Red-Horse and Siekmann, 2019), and with the recently characterized (mixed) capillary-arterial phenotype of endothelial cells (Kalucka et al., 2020).

Capillary arterialization is a kind of vascular remodeling process (Renna et al., 2013; Ogawa et al., 2018; Spallazzi et al., 2019). It is driven by the shear stress, pursuing to maintain an adequate cerebral blood flow (CBF) (Jung et al., 2017). Interestingly, successful treatments to improve CBF was not in correlation with ‘sprouting angiogenesis’ but with ‘collateral circulation’ (Korpisalo et al., 2011). Patients with a high reserve of collateral circulation were less vulnerable to cardiac infarcts and cerebral stroke (Menon et al., 2013; Jung et al., 2017). This point is important, because hippocampal vasculature shows great variability between individuals (Marinković et al., 1992), and between its own CA1-CA4 sub-regions (Ogawa et al., 2018).

Our approach showed some limitations. First, the procedure relies on the information in the databases. Hence, it is exposed to the same sources of variability between methods, protocols, and laboratories that the experimental data feeding these repositories. Second, as data came from a homogenate, the method cannot discern vascularization differences between the hippocampal subregions (CA1-CA4). Although we did not perform a segmented analysis of the vascular bed, we could detect the arterialization process in the Aged Group. Moreover, it was reported that non-segmented analysis yielded 69% more DEGs than the segmented analysis, sharing only 46% of the DEGs (Zhao et al., 2020). So, it could be beneficial to complement both types of analyses. Third, we decided to focus on the subset of genes that, belonging to endothelium, admits splice variants. This might result in the exclusion of some genes. As counterpart, the method showed enough capacity to capture distinct biologically relevant trends. Fourth, although we focus herein on endothelial cells alone, this type of study can be extended to all the components of the NVU. Fifth, considering that our results are based on transcriptional data, all the conclusions reported herein should be confirmed by other methods. Actually, the approach can also be extended to include micro-RNAs, circ-RNAs, lnc-RNAs, and proteomics if data were available.

## MATERIALS AND METHODS

### Data

Human hippocampal microarray data by Affymetrix HG133 plus 2 was gathered by Cotman's group (Berchtold et al., 2008, 2014b). To apply our computational pipeline, we retrieved these data from the Gene Expression Omnibus database (accession code: GSE11882, www.ncbi.nlm.nih.gov/geo). Details about the criteria of inclusion/exclusion, race, gender, causes of death, ApoE genotype, scores at MMSE and CDR tests, and histological Braak staging were reported in the Supplementary Materials of the original articles. We re-stratified the data from *n*=55 hippocampal samples by considering 64 years-old as cut-off value. Three groups were defined [healthy-young adults (Younger Group), healthy-elder individuals (Aged Group), and elderly patients with mild cognitive impairment (MCI group)]. Importantly, these data sets comprised balanced groups of men and women, matched by age. Hence, Group 1 comprised of nine men (age median=28 years; age range=20–45 years) and nine women (age median=44 years, age range=26–64 years), Group 2 included 15 men (age median=83 years, age range=69–97 years) and 14 women (age median=82.5 years, age range=70–99 years), while Group 3 was made up by four men (age median=86.5 years; age range=75-89 years) and four women (age median=88.5 years, age range=88–90 years).

### Computational methods

**Fig. 5.**
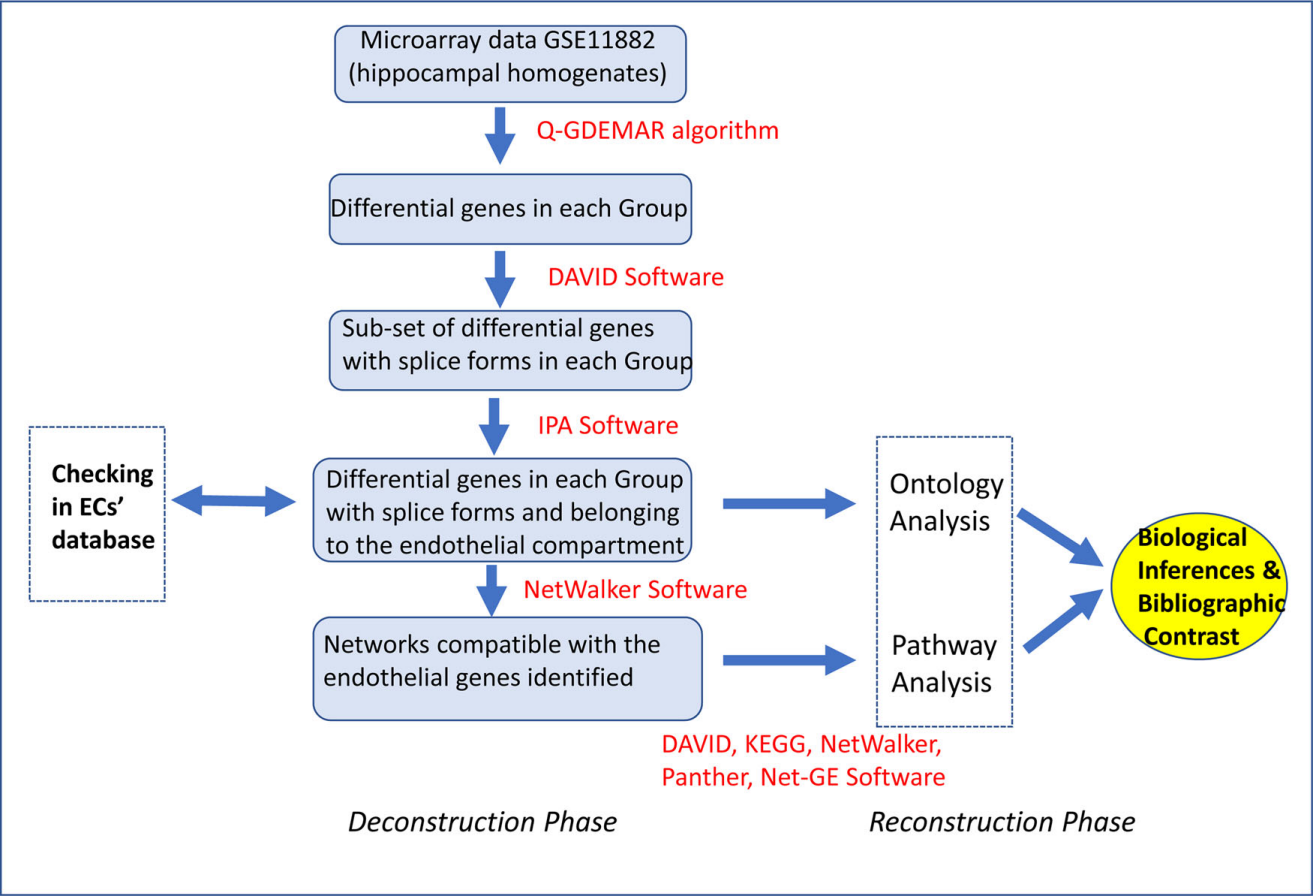
**Sequence of computational operations performed to deconstruct the global microarray data corresponding to samples of the hippocampal homogenates (GSE11882, GEO database), followed by the reconstruction of some plausible functional profiles that would be operating in the endothelium compartment of human hippocampus.** The control of the results included two aspects: (i) the checking of the genes identified by our computation pipeline against two reported independent data sets (Zhang et al., 2014; Munji et al., 2019); (ii) by comparing most of the inferences made from our analyses against some of the extensive list of references cited.

By a multi-step computational pipeline, the micro-array data corresponding to a total homogenate of human hippocampus was transformed into a set of data corresponding to the endothelium fraction of this tissue. This computational dissection, allowed us the re-analysis of the recovered data to establish some profiles of genes that might be operating in the endothelial compartment of the human hippocampus (see Fig. 5).

As is depicted in Fig. 5, we first determined the total differential genes in each defined group by means of our optimized post-processing method Q-GDEMAR (Quantile-Gaussian Deconvolution of Microarrays). This algorithm has been previously validated (Guebel et al., 2016) and used with several working systems (Guebel and Torres, 2016, 2018). Thereafter, a double filtering procedure was additionally applied. A first one was employed to perform an ontology analysis by using DAVID 6.8 software (Huang et al., 2009). This allowed us to identify which of the total differential genes admit alternative splice forms. The sub-sets of differential genes associated to splice forms were in turn subjected to a new screening to determine which of them actually occur in endothelial cells. This was done using the Ingenuity system (IPA™, Qiagen, USA). IPA™ also provides some relevant networks based on these molecules. The information obtained, although very rich, often resulted very heterogenous, because very distinct levels of information are mixed up in a same network. For this reason, the list of molecules arising from the Ingenuity system, were run again, this time on the NetWalker software (Komurov et al., 2012). This program not only adds connector molecules but allows discerning among distinct levels of action (genetic, protein–protein interactions, etc.). Finally, the arising list of molecules was subjected to a new ontology analysis at the level of biological processes and pathway analysis using several software packages as DAVID (Huang et al., 2009), NetWalker (Komurov et al., 2012), Net-GE (Bovo et al., 2016), KEGG (Kanehisa et al., 2017), and Panther (Mi et al., 2017), and DisGeNET (Piñero et al., 2020).

### Checking of gene identification

To assess the accuracy of our bioinformatic pipeline, we have tested whether the differential genes recovered as output of the computational procedure belong to the endothelial compartment. This was achieved by comparing the list of identified genes against two independent reported sets of data. One is the data-set generated by Munji et al. (2019) (GEO database, accession number GSE95401). The other is the data-set generated in the Barres & Wu's laboratories (Zhang et al., 2014), accessible from the so-called ‘Brain Cell RNAseq Browser’ (http://jiaqianwulab.org/braincell/RNASeq.html).

## Supplementary Material

Supplementary information
