# Supplementary material for: Mapping the transcriptomic changes of endothelial compartment in human hippocampus across aging and mild cognitive impairment
Source: Biol Open. 2021 May 24;10(5):bio057950. doi: 10.1242/bio.057950 (PMC8181899; doi:10.1242/bio.057950)
Supplement: Supplementary information [file biolopen-10-057950-s1.pdf]

Supplementary Information

Table S1. Main differential functionalities in the endothelial vascular compartment in human hippocampus arising from the ontology analysis (biological process) across the three groups of individuals assessed (Younger Group: Healthy-Adults; Aged Group: Healthy-Elder Adults; MCI Group: Elder Individuals with Mild Cognitive Impairment). Values are expressed as $-log_{10}$(FDR).

Functionality	Younger	Aged	MCI
BBB	2,59	2,06	1,70
BBB (Negative)	1,96	0,00	0,00
Artery development	2,55	6,90	5,35
Venous vessel morphogenesis	2,49	1,75	11,57
Mesenchymal differentiation	1,91	6,83	5,27
Abnormal Pericyte Morphology	0,00	2,77	0,00
Vasculogenesis/Vessel development	24,01	11,75	3,20
Vasculogenesis (Negative Reg.)	0,00	1,78	2,46
Endothelium development	3,30	2,75	4,42
Angiogenesis	20,31	7,83	3,10
Angiogenesis (Negative Reg.)	0,00	1,91	0,00
Sprouting Angiogenesis	6,95	2,92	5,95
Branching Morphogenesis	4.10	5,70	6,12
Angiopoietin/Tie2	3,97	1,87	3,60
Endothelium proliferation	5,87	5,06	2,96
Endothelium differentiation	9,87	3,62	3,16
Endothelium migration	4,31	2,79	6,09
Endothelial cell chemotaxis	2,47	1,60	2,28
Endothelial cell adhesion (homotypic/matrix)	3,27	1,48	1,51
Endothelium maturation	1,31	1,67	1,46
Smooth muscle proliferation	3,28	6,54	2,17
Weibel-Palade body	0,00	2,40	0,00

Biology Open • Supplementary information

Functionality	Younger	Elder	MCI
Circulatory systems	11,37	2,75	7,03
Blood pressure	2,08	2,71	1,74
Blood pressure (Negative Reg.)	2,59	2,06	0,00
Nitric Oxide	2,58	2,71	1,70
Nitric Oxide (Negative Reg.)	0,00	2,06	0,00
Cholesterol (storage, efflux, transport)	0,00	1,70	1,45
Lipoproteins metabolism	0,00	1,71	1,70
Lipoproteins (Negative Reg.)	0,00	2,33	0,00
Lactosylceramide/galactolipids/Gangliosides	0,00	0,00	1,79
Iron ion homeostasis	0,00	1,89	1,58
Detoxification	1,93	0,00	0,00
Sphingosin-1-P	2,11	0,00	0,00
Response to nitrite	0,00	1,45	0,00
Response to lipids	4,55	2,06	7,69
Response to Calcium	1,86	0,00	0,00
Calcium homeostasis	2,07	1,43	1,48
Inositosides	2,60	2,98	1,55
Inositosides (Negative Regulation)	0,00	1,87	0,00
NF-KappaB	1,42	2,56	1,90
Inflammation	7,43	15,94	8,35
Inflammation (Negative)	1,20	0,00	0,00
Accute Inflammation	1,43	2,32	1,92
Cytokine Production	2,57	4,47	3,22
Chemokine (Positive Regulation)	3,03	2,30	2,02
Chemokine (Negative Regulation)	1,45	1,45	0,00
Prostanoid Production	1,92	3,16	1,56
Thromboxan A2 receptor	6,47	1,35	5,05
Response to steroids	2,47	2,17	2,10
Icosanoids	0,00	1,75	0,00
Estrogens	2,29	4,68	1,63
Corticosteroids	1,79	2,95	6,46
Interferon type gamma	2,09	1,94	1,55
Other Interferons (I, III, alpha, beta)	0,00	1,63	1,42
Response to TGF	1,97	7,41	2,10
LTGF	0,00	1,50	0,00
TGF beta regulation	1,97	1,91	1,51
TGF beta (Negative Regulation)	1,35	2,29	1,44
TNF	2,12	1,47	4,17
Notch	1,63	2,20	2,10
Notch (Negative Regulation)	1,85	0,00	0,00

Biology Open • Supplementary information

Functionality	Younger	Elder	MCI
Response to growth factors	9,87	7,62	8,50
VEGF	2,58	2,69	2,46
HGF	1,96	1,77	2,08
EGF	1,96	0,00	1,17
PDGFR	2,43	2,15	1,57
FGF	0,00	1,57	2,11
FGF (Negative)	0,00	1,73	0,00
Corticotropin-realising factor	0,00	0,00	1,82
Canonical Wnt	1,05	1,85	1,88
Non-canonical Wnt	1,43	0,00	0,00
Non-canonical Wnt (Negative)	1,40	0,00	0,00
Insulin	2,11	1,31	2,14
Insulin (Negative)	1,68	0,00	0,00
Insulin Sensitivity	5,44	2,00	1,93
IGF	1,62	0,00	2,14
IL-1	1,64	2,02	4,00
IL-2	1,70	1,74	0,00
TIE	0,00	1,43	0,00
IL-4	1,62	0,00	0,00
IL-4 (Negative Regulation)	0,85	0,00	0,00
IL-5	0,00	1,68	0,00
IL-5 (Negative Regulation)	1,35	0,00	0,00
IL-6	0,00	2,79	2,73
IL-8	0,00	2,74	0,00
IL-12	1,37	2,33	1,55
IL-13	0,00	2,13	0,00
IL-13 (Negative Regulation)	1,45	0,00	0,00
IL-15	0,00	0,00	1,36
IL-18/-21/-23/-27/-35	0,00	0,00	1,41
GM-CSF	0,00	1,78	2,27

Biology Open • Supplementary information

Table S2: Detail of genes involved in the ontology functionalities of the Younger Group

Click here to download Table S2

Table S3: Detail of genes involved in the ontology functionalities of the Aged Group

Click here to download Table S3

Table S4: Detail of genes involved in the ontology functionalities of the MCI Group

Click here to download Table S4

Table S5: Pathway-based ontology comparison between Younger and Aged Groups

Click here to download Table S5

Biology Open • Supplementary information
